# Radiation therapy and PD-1/PD-L1 blockade: the clinical development of an evolving anticancer combination

**DOI:** 10.1186/s40425-018-0361-7

**Published:** 2018-06-04

**Authors:** Jun Gong, Thang Q. Le, Erminia Massarelli, Andrew E. Hendifar, Richard Tuli

**Affiliations:** 1 Department of Medical Oncology, City of Hope National Medical Center, Duarte, CA USA; 2 Division of Angiography and Interventional Radiology, Brigham and Women’s Hospital, Harvard Medical School, Boston, MA USA; 3 Division of Medical Oncology, Department of Medicine, Samuel Oschin Comprehensive Cancer Institute, Cedars-Sinai Medical Center, Los Angeles, CA USA; 4 Departments of Radiation Oncology and Biomedical Sciences, Samuel Oschin Comprehensive Cancer Institute, Cedars-Sinai Medical Center, 8700 Beverly Blvd, AC 1023, Los Angeles, CA 90048 USA

**Keywords:** Radiation therapy, PD-1, PD-L1, Clinical trials, Preclinical, Antitumor, Immune response, Checkpoint inhibitor

## Abstract

Several inhibitors of programmed cell death-1 (PD-1) and programmed death ligand-1 (PD-L1) have been approved as a form of immunotherapy for multiple cancers. Ionizing radiation therapy (RT) has been shown to enhance the priming and effector phases of the antitumor T-cell response rendering it an attractive therapy to combine with PD-1/PD-L1 inhibitors. Preclinical data support the rational combination of the 2 modalities and has paved way for the clinical development of the combination across a spectrum of cancers. In this review, we highlight the preclinical and clinical development of combined RT and PD-1/PD-L1 blockade to date. In addition to a comprehensive evaluation of available safety and efficacy data, we discuss important points of consideration in clinical trial design for this promising combination.

## Background

Early preclinical evidence demonstrated that activation of the programmed cell death 1 (PD-1) and programmed death ligand 1 (PD-L1) axis suppressed the activation and proliferation of tumor antigen-specific T-cells and promoted tumorigenesis [1, 2]. These processes were reversed with PD-1/PD-L1 blockade and supported the concept of PD-1/PD-L1 blockade as a potential form of anti-cancer immunotherapy. The first agents in the family of PD-1/PD-L1 inhibitors to be approved by the Food and Drug Administration (FDA) were the humanized monoclonal IgG4 antibodies, pembrolizumab and nivolumab, that targeted PD-1 in unresectable or advanced melanoma [3–10]. There are currently 5 PD-1/PD-L1 inhibitors approved by the FDA for the treatment of a number of solid tumors and hematologic malignancies [11–43].

**Fig. 1:**
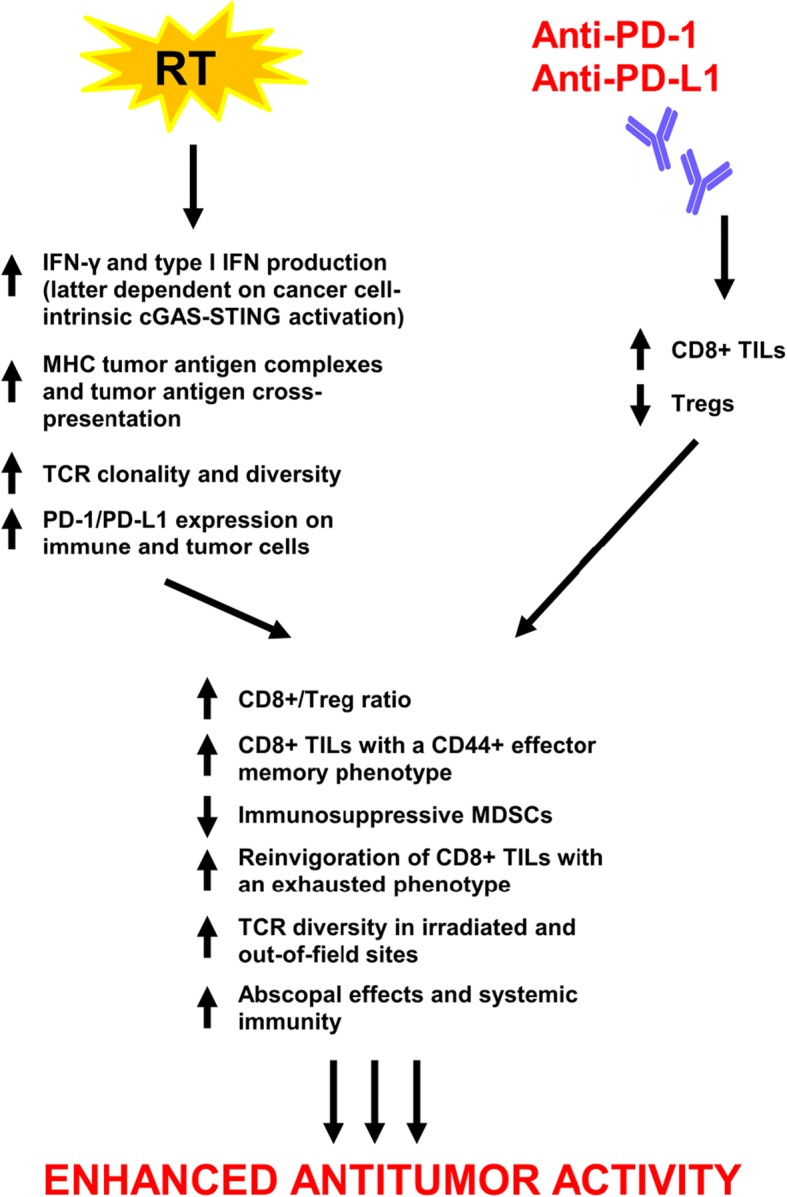
Proposed mechanisms of synergy between RT and PD-1/PD-L1 inhibitors. Emerging evidence demonstrates that immune modulation from PD-1/PD-L1 inhibitors and RT through nonredundant pathways contributes to synergistic antitumor activity, thereby forming the basis for the rationale combination of the two modalities. RT, radiation therapy; PD-1, programmed cell death 1 receptor; PD-L1, programmed death ligand 1; IFN-γ, interferon-γ; cGAS, cyclic GMP-AMP (cGAMP) synthase; STING, stimulator of interferon genes; MHC, major histocompatibility complex; TCR, T-cell receptor; TILs, tumor-infiltrating lymphocytes, Tregs; regulatory T cells; MDSCs, myeloid-derived suppressor cells

Ionizing radiation therapy (RT) is widely used in the definitive and metastatic setting for local tumor control; however, the ability of radiation to elicit a systemic tumor response with associated regression of untreated metastases outside of the radiation field has been reported and was first described as the abscopal effect [44]. Increasing evidence supports that the abscopal effect is likely immune-mediated – largely, in a T-cell dependent manner with a complex interplay between proimmunogenic and proinflammatory factors [45–53]. Over time, recognition of the immunomodulatory properties of radiation has led to the integration of RT with immune-modulating agents including immune checkpoint inhibitors to potentially develop a combination therapy with enhanced or synergistic anticancer activity (Fig. 1).

Indeed, an initial preclinical study showed that combining RT (1–2 fractions of 12 Gray (Gy) to the primary tumor) with an anti-cytotoxic T lymphocyte-associated antigen-4 (CTLA-4) monoclonal antibody resulted in synergistic antitumor activity in a poorly immunogenic metastatic mammary carcinoma mouse model when CTLA-4 blockade by itself was ineffective [54]. Enhanced antitumor responses have also been observed across several preclinical animal models treated with combined RT and CTLA-4 blockade [55–58]. Since the first preclinical studies that highlighted the synergistic antitumor activity of combination RT and CTLA-4 blockade, several prospective clinical trials have reported on the activity of RT and ipilimumab in advanced solid tumors [59–66]. Similarly, there are numerous ongoing clinical trials investigating the combination of RT and CTLA-4 blockade that have been extensively reviewed and are beyond the scope of this manuscript [67, 68]. Herein, we review in detail the preclinical and clinical development of the combination of RT and PD-1/PD-L1 inhibitors in cancer therapy.

## Preclinical studies

### The efficacy of combination RT and checkpoint blockade is associated with modulation of immune parameters within the tumor microenvironment

Early investigations in mouse models of solid and hematologic malignancies showed enhanced antitumor effects when treated with PD-1 or PD-L1 blockade in combination with in-field RT, sublethal total body irradiation (TBI), or stereotactic radiosurgery (SRS) compared to single modality treatment (Table 1) [69–85]. Combined modality therapy was associated with higher levels of CD8+/interferon-γ (IFNγ)+/tumor necrosis factor-α (TNFα) + cytotoxic T-cells, increased PD-1, T-cell immunoglobulin mucin-3 (TIM-3), lymphocyte-activation gene 3 (LAG-3), and 2B4 (immune checkpoints) expression on CD8+ T-cells, decreased numbers of CD4+/FOXP3+ regulatory T-cells (Tregs) and myeloid-derived suppressor cells (MDSCs), upregulation of PD-L1 on dendritic cells and tumor cells in irradiated tumors, RT-induced upregulation of major histocompatibility complex (MHC) class I tumor-associated antigen complexes, and enhanced antigen cross-presentation in draining lymph nodes compared to single modality arms [71, 72, 74, 76–79].

**Table 1:** Preclinical studies demonstrating antitumor activity of combined radiation therapy and PD-1/PD-L1 blockade

Cell line	Experimental model	RT dose	PD-1/PD-L1 dose	Ref.
B16-D5 (melanoma)	Mice subcutaneous	TBI 600 cGy (1 fraction)	PD-L1 mAb 20 mg/kg IP starting on day 4 then every 3–4 days +1X10^6^ gp100 or OVA_257–264_ pulsed dendritic cell vaccine SC on day 4 and 11 ± 1X10^7^ pmel T-cells (adoptive transfer) IV on day 4 after inoculation	[69]
AT.3 (triple-negative mammary)	Mice xenograft	12 Gy (1 fraction) or 4–5 Gy (4 fractions)	PD-1 mAb 100 μg + CD137 mAb 100 μg IP on days 0, 4, 8, and 12 of RT	[70, 71]
GL261 (glioma)	Mice xenograft	10 Gy (1 fraction)	PD-1 mAb 10 mg/kg IP on days 10, 12, and 14 of RT	[72]
B16-SIY (melanoma) TUBO (mammary)	Mice subcutaneous	25 Gy (2 fractions) 15 Gy (1 fraction)	PD-L1 mAb 200 μg IP every 3 days for 4 doses starting 3 weeks after RT	[92]
5 T33 (myeloma) A20 (B-cell lymphoma) C1498 (leukemia)	Mice intravenous	TBI 500 cGy (1 fraction)	PD-L1 mAb 200 μg IP on days 12, 14, 17, 19, 21, 26, and 28 after inoculation	[75]
5 T33 (myeloma)	Mice intravenous	TBI 1100 cGy (1 fraction)	HSCT on day 0 + PD-L1 mAb 200 μg IP on days 3, 5, 10, 12, 17, and 19 after HSCT ± vaccine (irradiated 5 T33 cells or 5 T33 cells transfected with empty vectors) on days 3, 10, and 17 after HSCT	[73]
5 T33 (myeloma)	Mice intravenous	TBI 500 cGy (1 fraction)	PD-L1 mAb 200 μg IP on days 12, 14, 17, 19, 21, 26, and 28 after inoculation ± LAG-3, TIM-3, or CTLA-4 mAbs 200 μg IP on same days	[74]
CT26 (colon 4434 (*BRAF*^V600E^-mutant melanoma) 4 T1 (triple-negative mammary)	Mice subcutaneous	10 Gy (5 fractions)	PD-1 or PD-L1 mAb 10 mg/kg IP 3 times weekly up to 3 weeks starting on day 1 of RT	[86]
TUBO (mammary) MC38 (colon	Mice subcutaneous	12 Gy (1 fraction)	PD-L1 mAb 200 μg IP every 3 days for 4 doses starting on day 0 or 1 of RT	[76]
TSA (mammary)	Mice subcutaneous	24 Gy (3 fractions)	PD-1 mAb (dose NR) starting on day 15 after inoculation and every 4 days thereafter	[77]
B16-OVA (melanoma) RENCA (renal)	Mice subcutaneous	15 Gy (1 fraction)	PD-1 mAb 10 mg/kg ± CTLA-4 mAb 10 mg/kg IP on days 7, 9, 11, 14, and 16 following tumor cell inoculation	[87]
B16-OVA (melanoma) 4 T1-HA (mammary)	Mice subcutaneous	12 Gy (1 fraction)	PD-1 mAb 200 μg IP every 3 days for 3 doses starting 1 day prior to RT	[79]
PyMT (mammary)	Mice subcutaneous	12 Gy (1 fraction)	PD-1 mAb dose NR + single dose of CTLA-4 mAb (dose NR) 3 days prior to PD-1 and RT	[78]
B16-F10 (melanoma)	Mice subcutaneous	20 Gy (1 fraction)	PD-L1 mAb 200 μg + CTLA-4 mAb 200 μg IP every 3 days for 3 doses starting 5 or 9 days after inoculation	[61]
Meer (head and neck squamous)	Mice subcutaneous	1, 6, 10 Gy fractions	PD-L1 antibody dose NR	[82]
Adeno-Cre viral vector (lung)	GEMM intrathoracic injection	8.5 Gy twice daily over 2 days	PD-1 mAb 200 μg IP 3 times weekly starting 6 h after second RT dose	[81]
MB49 (bladder)	Mice xenograft	12 Gy (1 fraction)	PD-L1 mAb 250 μg IP twice weekly for 4 doses starting 1 day prior to RT	[84]
MC38 (colon 4 T1 (mammary) B16F10-OVA (melanoma)	Mice subcutaneous	24 Gy (3 fractions)	PD-1 mAb ± CD137 mAb 5–10 mg/kg IP on days 13, 15, and 17 after inoculation	[83]
4-hydroxytamoxifen induction (*BRAF*-mutant, *PTEN*-deficient melanoma)	GEMM topical induction	14 Gy (1 fraction)	PD-1 + CD137 or PD-1 + CTLA-4 mAb 100 μg IP twice weekly for 4 doses on day 1 of RT	[99]
344SQ (lung)	Mice subcutaneous	36 Gy (3 fractions)	PD-1 mAb 10 mg/kg IP starting on day 1 of RT and continued for additional 3–4 doses	[91]
ARK (esophageal squamous)	Mice subcutaneous	20 Gy (10 fractions)	PD-1 mAb (dose NR) starting 2 days before RT and every 3 days thereafter ± carboplatin and paclitaxel IP (dose NR) on day 1 of RT and every 3 fractions	[85]
GL261 (glioma)	Mice xenograft	10 Gy (1 fraction)	PD-1 mAb 200 μg IP on days 10, 12, and 14 of RT ± TIM-3 mAb 250 μg IP days 7, 11, and 15 of RT	[90]
CT26 (colon 4434 (*BRAF*^V600E^-mutant melanoma)	Mice subcutaneous	10 Gy (5 fractions)	PD-1 or PD-L1 mAb 10 mg/kg IP 3 times weekly for 1 week starting on day 1 of RT	[88]
TSA (mammary)	Mice subcutaneous	24 Gy (3 fractions) on days 12, 13 and 14 after inoculation	PD-1 mAb 200 μg IP on days 12, 15, 19, 22 and 26 after inoculation	[89]
Hep-55.1c (hepatocellular)	Mice orthotopic	30 Gy (3 fractions)	PD-1 mAb 250 μg IP on days 7, 14, and 21 after inoculation	[96]
KPC and Pan02 (pancreatic)	Mice subcutaneous	6, 12, or 20 Gy (1 fraction) 10 Gy (5 fractions) 15 Gy (5 fractions)	PD-L1 mAb 10 mg/kg IP on days 4, 7, 10, and 13 after inoculation + gemcitabine 100 mg/kg IP on days 0 and 3 of inoculation	[95]
HCa-1 (hepatocellular)	Mice intramuscular	10 Gy (1 fraction)	PD-L1 mAb 10 mg/kg IP every 3 days for 4 doses starting on day 1 of RT	[97]
LM8 (osteosarcoma)	Mice subcutaneous	10 Gy (1 fraction)	PD-L1 mAb 150 μg + CTLA-4 mAb 150 μg IP every 3 days for 3 doses starting on days 9, 12, and 15 after inoculation	[98]
CT26 (colon	Mice intradermal	RFA 17-gauge single ablation electrode for 3.5–4.5 min at target temperature of 70 degrees C	PD-1 mAb 200 μg IP every 3 days for 4 doses	[94]

*RT* radiation therapy, *TBI* total body irradiation, *cGy* centigray *mAb* monoclonal antibody, *IP* intraperitoneal, *SC* subcutaneous, *IV* intravenous, *Gy* Gray, *HSCT* hematopoietic stem cell transplantation, *LAG-3* lymphocyte-activation gene 3, *TIM-3* T-cell immunoglobulin mucin-3, *NR* not reported, *GEMM* genetically engineered mouse model, *RFA* radiofrequency ablation

### Combination modality-induced immune profile changes may be time-dependent

Early syngeneic mouse tumor models demonstrating significant improvements in survival and tumor volume reduction with the combination of RT and PD-1 or PD-L1 blockade compared to single modality and control arms identified elevations in tumor cell PD-L1 expression that were CD8+ T-cell and IFNγ-dependent following irradiation (10 Gy over 5 daily fractions) compared to non-irradiated mice with peak levels occurring 72 h after last dose of RT [86]. RT-induced increases in the CD8+/Treg ratio and PD-L1 expression occurred 24–96 h post-RT in a separate mouse model [81]. In colon carcinoma tumors, the addition of PD-L1 blockade on day 1 of RT (schedule A), day 5 of RT (schedule B), or 7 days after RT (schedule C) showed that there was no significant difference in overall survival (OS) between schedule A and B (*p* > 0.05) though sequential therapy (schedule C) was ineffective in enhancing OS compared to RT alone (median OS 30 days vs. 35 days, *p* > 0.05) [86]. Notably, PD-1 expression was significantly decreased on CD8+ T-cells 7 days after RT compared to time-matched controls (*p* < 0.05).

### Abscopal effects and systemic immunity

On subcutaneous tumor flank rechallenge of treatment-naïve mice and mice cured by combination RT and checkpoint blockade, immunologic memory was established in cured mice but not in treatment-naïve mice suggesting that the immune system in cured mice retained the ability to recognize tumor-associated antigens and mount an immune response of greater magnitude and speed upon rechallenge, i.e., systemic immunity [71, 72]. Abscopal effects have been shown to be mediated, in part, by PD-1 as administration of a single fraction of 15 Gy by stereotactic ablative radiotherapy (SABR) to the primary tumor in a melanoma subcutaneous mouse model resulted in significant reduction in tumor volumes of secondary nonirradiated tumors in PD-1-knockout mice compared to PD-1-wild-type (WT) mice [87]. Addition of a PD-1 inhibitor to SABR resulted in synergistic antitumor activity on the primary tumor compared to PD-1 inhibitor or SABR alone and recapitulated abscopal effects on secondary nonirradiated tumors in PD-1-WT mice when treatment alone with anti-PD-1 or SABR did not reduce secondary tumor growth. Furthermore, following RT, higher levels of PD-1+ CD11a^high^ CD8+ T-cells were seen in primary tumors compared to secondary tumors and higher levels in irradiated compared to nonirradiated tumors; this population of cells appeared to comprise the principal tumor-specific reactive phenotype. This latter finding has been confirmed in another study where RT increased T-cell receptor (TCR) repertoire clonality and diversity of the TCR repertoire in irradiated tumors compared to controls, however, the addition of PD-1 inhibition to RT increased TCR diversity both in irradiated and out-of-field sites [88]. Further analysis revealed that most of these TCR clones arose from progenitor clones that were established in tumors prior to therapy, and it is the influx of tumor-infiltrating lymphocytes (TILs) from outside the tumor along with resident-tumor infiltrating T-cells that contribute to the enhanced tumor responses seen with combination therapy.

Recently, durable regression of irradiated tumors and abscopal responses observed in mammary tumor-bearing mouse models treated with combination RT and checkpoint blockade were shown to be dependent on cancer cell-intrinsic activation of the type I IFN pathway as mediated by cyclic GMP-AMP (cGAMP) synthase (cGAS) and stimulator of interferon genes (STING) signaling [89]. RT-induced abscopal responses with PD-1 blockade were additionally shown to be regulated by *Trex1* where induction of Trex1 expression in cancer cells resulted in loss of abscopal responses in mice treated with the combination.

### Combined modality therapy reverses T-cell exhaustion and resistance to RT and anti-PD-1 therapy

Murine tumor xenografts have shown that increasing levels of PD-1 and TIM-3 co-expression in CD4+ T-cells, CD8+ T-cells, and Tregs over time contribute to an exhausted or impaired T-cell phenotype [90]. Furthermore, resistance to anti-PD-1 therapy in RT-refractory tumors has been characterized by significant elevations in expression of genes associated with T-cell exhaustion, increased levels of checkpoints including LAG-3, TIM3, and CTLA-4 on CD4+ T-cells, and decreased number of CD11c + tumor-associated macrophages (TAMs) [81]. The addition of immune checkpoint inhibitors to RT has been shown to enhance tumor response compared to controls across several mouse tumor models through reinvigoration of exhausted CD8+ TILs characterized by increased Ki67+ GzmB+ T-cells within the exhausted PD-1+ Eomes+ T-cell pool, increased CD8+ CD44+ TILs, and increased CD8+/Treg ratio [61, 77, 85].

Moreover, an anti-PD-1-resistant murine lung cancer model established through sequential in vivo passage of nonresponsive tumors to ongoing anti-PD-1 therapy was characterized by significant downregulation of MHC class I and II genes including β2-microglobulin and reduction in CD4+/CD8+ TILs and IFN-γ production in resistant tumors compared to parental tumors [91]. Addition of RT induced IFN-γ production and MHC class I expression and ultimately restored response to PD-1 blockade in resistant tumors. Addition of a PD-L1 inhibitor has been shown to reverse RT-induced tumor equilibrium in favor of tumor regression in mice subcutaneously injected with melanoma and breast tumors demonstrating RT-induced stable disease (SD, defined as ≥3 weeks) characterized by a transient rise and fall in levels of tumor-infiltrating CD8+ T-cells and IFNγ [92]. Extrinsic RT resistance has been recently shown to be contributed by RT-induced host STING activation resulting in immunosuppressive MDSC recruitment that is mediated by chemokine receptor type 2 (CCR2) in a syngeneic mouse model of colon carcinoma [93]. Treatment with anti-CCR2 antibodies could potentially serve a role in reversing RT resistance by attenuating host STING-mediated immunosuppression and complement RT and checkpoint blockade combinations.

A growing body of preclinical evidence supports the combination of other immunotherapeutic agents with RT or radiofrequency ablation (RFA), immune checkpoint blockade, and/or chemotherapy to enhance tumor growth control (and often systemic control)in preclinical mouse models; synergistic antitumor activity with multimodality therapy was characterized by tumor cell PD-L1 expression in a JAK/Stat1-dependent manner and reduced numbers of CD11b + Gr1+ cells (MDSCs) [90, 94–99].

### Toxicities

Several preclinical studies have investigated the toxicity of combined RT and checkpoint blockade. Notably, one investigation of lung-irradiated (20 Gy) C57bl/6-WT mice receiving anti-PD-1 antibody (10 mg/kg intraperitoneal twice per week for 5 doses) showed more findings of abnormal alveoli, inflammatory changes, and exudates in the alveolar septa associated with a 2.1-fold increase in CD8+ T-cells in the irradiated lung tissues of mice in the RT and PD-1 blockade arm though post-RT mortality up to 120 days was not significantly different in the RT alone vs. RT and PD-1 blockade arm (*p* = 0.657) [100]. A separate study, however, using a similar dose of 20 Gy of thoracic RT (designed to induce mortality) to C57bl/6 mice identified worse survival with RT and PD-1 blockade (36% survived) than RT alone (70% survived, *p* = 0.0169) at 21 days post-RT and increased T-cell infiltrates in lung and cardiac tissues (both in- and out-of-field) of mice treated with RT and PD-1 blockade compared to RT alone putatively due to enhanced healthy tissue damage by T-cell activation with the addition of PD-1 blockade to thoracic RT [101]. Incorporating PD-1 blockade to cardiac RT in mice has also shown to decrease survival and exacerbate cardiac dysfunction and myocarditis that are CD8+ T-cell-mediated [102].

## Clinical studies

### Retrospective studies

Numerous case reports and case series have documented clinically significant, and often durable, tumor responses to the combination of RT and PD-1/PD-L1 blockade in advanced or metastatic melanoma, NSCLC, Hodgkin lymphoma, RCC, and cervical cancer [103–112]. Initial retrospective series of patients with melanoma brain metastases treated with SRS or fractionated RT within 3–6 months of receiving anti-PD-1 therapy produced promising 1-year OS rates and significantly improved 6- and 12-month distant brain metastasis control and OS rates in those treated with SRS and anti-PD-1 therapy vs. SRS and chemotherapy (Table 2) [113, 114]. In 24 patients with brain metastases from melanoma (54%) and NSCLC (46%), treatment with SRS before, during, or after PD-1 blockade produced 6- and 12-month median OS rates of 85 and 78%, respectively [115]. One retrospective study investigated 53 patients with metastatic melanoma treated with RT sequential or concurrent to anti-PD-1 therapy or as salvage therapy in the setting of progression on anti-PD-1 therapy (35 patients received extracranial RT or intracranial SRS and 21 patients received whole brain radiotherapy (WBRT)) and showed that median OS and ORR were not significantly different between concurrent and sequential RT/SRS cohorts (Table 2) [116].

**Table 2:** Retrospective clinical studies with available results on the antitumor activity of combined radiation therapy and PD-1/PD-L1 blockade

Study	n	Design	Outcomes	Toxicities	Ref.
RS	26	Melanoma BMs treated with SRS or FSRT (16–30 Gy X 1–5 fractions) within 6 mo of nivolumab (1, 3, or 10 mg/kg every 2 weeks for 12 doses then every 12 weeks for 8 doses)	Median OS 11.8 mo (range 0.5–33.9) and 1-year OS 55% in unresected BMs; median OS not reached and 1-year OS 100% in resected BMs	1 grade 2 headache relieved with steroids	[114]
RS	96	Melanoma BMs treated with SRS (majority 24 Gy X 1 fraction) within 3 mo of nivolumab 3 mg/kg every 2 weeks, pembrolizumab 2 mg/kg every 3 weeks, or other systemic therapies	6- and 12-mo distant BM control rate 61%/38% anti-PD-1, 26%/21% anti-CTLA-4, 53%/20% BRAF/MEK inhibitor, 15%/5% chemotherapy (*p* = 0.008); 6- and 12-mo OS 81%/66% anti-PD-1, 84%/50% anti-CTLA-4, 83%/75% BRAF/MEK inhibitor, 70%/15% chemotherapy (*p* = 0.004)	For anti-PD-1 therapy: 1 grade 2 headache managed with steroids	[113]
RS	24	Melanoma and NSCLC BMs treated with SRS (median 20 Gy/fraction, IQR 16–21) within median 19 weeks (range 0–107) of nivolumab or pembrolizumab (median 5 cycles, IQR 3–6)	6- and 12-mo OS 85 and 78%; median OS not reached; 6- and 12-mo distant brain progression rate 37 and 65%	2 patients grade ≥ 3 CNS toxicity: 1 seizure and 1 symptomatic radionecrosis requiring surgery	[115]
RS	53	Metastatic melanoma treated with extracranial RT/intracranial SRS (8–30 Gy X 1–10 fractions) or WBRT (median 30 Gy X10 fractions) and pembrolizumab 2 mg/kg every 3 weeks or nivolumab 3 mg/kg every 2 weeks as concurrent, sequential, or salvage (following progression on anti-PD-1 therapy) therapy	Medians OS 6.4 vs. 8.6 mo (*p* = 0.7672) for concurrent vs. sequential RT/SRS; ORR 31% vs. 36% (*p* = 1) for concurrent vs. sequential RT/SRS; lesional response rate 45% for 30 progressing lesions treated with salvage RT/SRS	For RT arm: 3 patients grade ≥ 3 rash, 1 grade ≥ 3 diarrhea, 2 grade ≥ 3 radiation dermatitis, 1 grade ≥ 3 radionecrosis; for WBRT arm: 1 grade ≥ 3 nausea, 1 grade ≥ 3 cognitive changes, 2 grade ≥ 3 rash	[116]
RS	75	Melanoma BMs treated with SRS (median 20 Gy, range 12–24 Gy) within ±4 weeks (concurrent) of pembrolizumab 2 or 10 mg/kg every 2–3 weeks or nivolumab 3 mg/kg every 2–3 weeks or ipilimumab	Median % lesion volume reduction at 3 mo (− 83.0% vs. -52.8%, *p* < 0.0001) and 6 mo (− 94.9% vs. -66.2%, *p* < 0.0001) for concurrent vs. noncurrent; median % lesion volume reduction at 3 mo (− 89.3% vs. -66.2%, *p* < 0.0001) and 6 mo (− 95.1% vs. -75.9%, *p* = 0.0004) for anti-PD-1 vs. anti-CTLA-4	NR	[117]
RS	21	Metastatic NSCLC treated with RT (8–30 Gy X 1–10 fractions) while receiving anti-PD-1, anti-PD-L1, and/or anti-CTLA-4, or other immune therapy	6- and 12-mo local control rates 91.7 and 85.2%; median time to systemic progression 2.3 mo (95% CI 1.0–4.5); median OS 7.2 mo (95% CI 4.2–11.1)	1 grade 4 cerebral edema (WBRT) and 1 grade 3 pneumonitis	[118]
RS	25	Unresectable melanoma treated with hypofractionated RT (1 weekly fraction over 4–5 weeks (84%) or 1 gammaknife RT for BMs (16%)) within 3 mo of anti-PD-1 (early) or > 3 mo after anti-PD-1 therapy (late)	CR, PR, SD, and PD rates for radiated sites 24, 8, 44, and 28% and for nonirradiated sites 29, 19, 19, and 33%, respectively; abscopal responses (CR or PR) in 56% for addition of late RT	No unusual AEs reported	[119]
RS	15	Metastatic melanoma, RCC, NSCLC treated with palliative RT (total 8–36 Gy via 3–8 Gy fractions) within ±75 days of PD-1 inhibitor	Safety analysis	All-grade immune-related AEs in 3 patients (20%) and 1 RT-related AE (7%) of moderate mucositis; no cases of pneumonitis	[123]
RS	84	Metastatic melanoma, NSCLC, and other solid tumors treated with thoracic RT (median total dose 3000 cGy (range 600–7400 X 10 fractions) within 1 month (concurrent) or up 6 months (sequential) of PD-1/PD-L1 and/or CTLA-4 blockade	No significant differences in toxicity rates between PD-1/PD-L1 and CTLA-4 inhibitors or concurrent and sequential treatment	For all-grade AEs: 6 patients with pneumonitis (7.2%, 1 grade ≥ 3); for grade ≥ 2 AEs: 14 fatigue, 9 rash, 10 GI toxicities, 12 infections, 8 thyroid dysfunction, 7 renal injury, and 9 other	[124]
RS	29	Metastatic NSCLC treated with thoracic RT (10–70 Gy X 1–35 fractions) within 6 mo of PD-1/PD-L1 and/or CTLA-4 blockade	Median PFS and OS of 3.8 mo (95% CI 1.9–8) and 9.2 mo (95% CI 5.1-not reached)	Possible treatment-related AEs: 1 grade 5 pneumonitis and 2 grade 3 pneumonitis	[125]
RS	133	Metastatic NSCLC, melanoma, and RCC treated with palliative RT (8–37.5 Gy X 1–15 fractions) within 180 days of PD-1 or CTLA-4 inhibitor	No significant difference in immune-related AEs between those receiving RT during/after checkpoint inhibitors and before checkpoint inhibitors (*p* = 0.053), receiving RT within 14 days or outside 14 days of checkpoint blockade (*p* = 0.06), and of site of irradiation	All-grade immune-related AEs: 20% dermatitis, 8% colitis, 5% transaminitis; grade ≥ 3 immune-related AEs: 4% colitis, 2% transaminitis, 2% hypophysitis	[127]
RS	137	Metastatic NSCLC, melanoma, and RCC treated with WBRT (12–39 Gy), SRS (15–30 Gy), or extracranial RT (8–66 Gy) within a median 85 days (IQR 34–181) of anti-PD-1 therapy	Median OS 249 days (IQR 90–689) following PD-1 blockade; on multivariate analysis HR for death 3.1 (95% CI 1.7–5.9) for NSCLC and HR 3.2 (95% CI 1.2–7.9) for RCC vs. melanoma (*p* = 0.0008)	No grade 4–5 immune-related AEs	[120]
RS	17	NSCLC BMs treated with SRS or FSRT (18–25 Gy X 1–5 fractions) within ±6 mo of nivolumab or durvalumab	Distant brain control rate 57% (RT during or before PD-1/PD-L1 blockade) vs. 0% (RT after, p = 0.05); median OS for SRS during/before PD-1/PD-L1 blockade vs. SRS after (HR 3.6, 95% CI 0.74–26.9, *p* = 0.11) on multivariate analysis	No neurologic/ cutaneous AEs with SRS and anti-PD-1/PD-L1 therapy (41% received prophylactic dexamethasone before SRS); 1 patient each discontinued PD-1/PD-L1 inhibitor due to colitis and pneumonitis	[128]
RS	137	Melanoma BMs treated with SRS or WBRT (median 20 Gy, range 12–30) within 1 year of PD-1 or CTLA-4 blockade	Median OS 16.9 mo; for radionecrosis: 37 patients (27%); no difference in risk between ipilimumab and pembrolizumab (*p* = 0.549) or CTLA-4 and PD-1 (*p* = 0.86); 1-year OS 78.4% vs. 55.06% (without radionecrosis, *p* = 0.341)	See outcomes	[129]
RS	98	Advanced NSCLC treated with palliative RT any time point before (median 9.5 mo, range 1–106) first cycle of pembrolizumab 2 or 10 mg/kg every 2–3 weeks	Any previous RT vs. no previous RT: median PFS 4.4 mo (95% CI 2.1–8.6) vs. 2.1 mo (95% CI 1.6–2.3, HR 0.56, 95% CI 0.34–0.91, *p* = 0.019); median OS 10.7 mo (95% CI 6.5–18.9) vs. 5.3 mo (95% CI 2.7–7.7, HR 0.58, 95% CI 0.36–0.94, *p* = 0.026)	All-grade treatment-related pulmonary toxicity in 3 patients (13%, with RT) vs. 1 (1% without RT, *p* = 0.046); grade ≥ 3 treatment-related pulmonary toxicity similar in both arms (1 each, *p* = 0.44)	[121]
RS	108	Melanoma BMs treated with SRS and/or WBRT (dose NR) within ±6 weeks of various systemic therapies	In combination with RT: median OS 7.5 mo with CTLA-4 (95% CI 4.4–15.6), 20.4 mo PD-1 (95% CI 8.8-NA), and 17.8 mo BRAF ± MEK inhibitor (95% CI 11.8-NA)	2 radiation necrosis (SRS + anti-PD-1) treated with surgery, steroids, and bevacizumab	[122]

*RS* retrospective study, *BMs* brain metastases, *SRS* stereotactic radiosurgery, *FSRT* fractionated stereotactic RT, *Gy* Gray, *OS* overall survival, *NSCLC* non-small cell lung cancer, *IQR* interquartile range, *CNS* central nervous system, *RT* radiotherapy, *WBRT* whole brain radiation therapy, *ORR* overall response rate, *NR* not reported, *CI* confidence interval, *CR* complete response, *PR* partial response, *SD* stable disease, *PD* progressive disease, *AEs* adverse events, *RCC* renal cell carcinoma, *GI* gastrointestinal, *HR* hazard ratio, *PFS* progression-free survival, *NA* not applicable

A single-institute retrospective trial analyzed the efficacy of concurrent SRS and anti-PD-1 or anti-CTLA-4 therapy (defined as SRS within 4 weeks of administration of checkpoint inhibitors) in 75 patients with melanoma brain metastases and identified significantly improved median percent reduction in lesion volume with concurrent compared to nonconcurrent arms and with anti-PD-1 compared to anti-CTLA-4 arms at 3 months and 6 months [117]. However, when both anti-PD-1 and anti-CTLA-4 therapies were combined there was no significant difference in median OS between nonconcurrent (9.0 months, range 2.1–61.8) and concurrent arms (19.1 months, range 2.7–64.2, *p* = 0.0691). In solely metastatic NSCLC patients (*n* = 21), combined RT to oligoprogressive sites along with PD-1/PD-L1 blockade or other immune therapies resulted in excellent local control, median time to systemic progression of 2.3 months (95% confidence interval (CI) 1.0–4.5), and median OS of 7.2 months (95% CI 4.2–11.1) [118]. Among 25 patients with unresectable melanoma, abscopal responses (CR or PR) were observed in 56% of patients with the addition of late RT (> 3 months of insufficient response to anti-PD-1 monotherapy) [119].

A group of 137 patients with metastatic melanoma, NSCLC, and RCC treated with WBRT, SRS, or extracranial RT before or after initiation of PD-1 blockade experienced a median OS 249 days (8 months; interquartile range (IQR) 90–689) following the start of anti-PD-1 therapy though OS was 25.7 months in the cohort receiving brain RT as first form of palliative RT [120]. On multivariate analysis, melanoma patients fared best as the hazard ratio (HR) for death was 3.1 (95% CI 1.7–5.9) for NSCLC and HR of 3.2 (95% CI 1.2–7.9) for RCC compared to melanoma (*p* = 0.0008) possibly due to improved responses to checkpoint inhibitors in melanoma with the incorporation of both PD-1 and CTLA-4 inhibitors into standard care.

A secondary analysis of the phase I KEYNOTE-001 trial of 98 patients with locally advanced or metastatic NSCLC treated with pembrolizumab showed significantly improved median OS of 10.7 months (95% CI 6.5–18.9) vs. 5.3 months (95% CI 2.7–7.7, HR 0.58, 95% CI 0.36–0.94, *p* = 0.026) in those who ever did and did not receive RT, respectively [121]. In spite of these interesting clinical results, no data are provided on the type, dose, schedule of radiotherapy or the tumor burden of patients receiving therapy making the results hard to interpret. Interestingly, one retrospective series of 108 patients with melanoma brain metastases treated with SRS and/or WBRT concurrently with various contemporary systemic therapies highlighted that RT in combination with anti-PD-1 therapy produced among the best OS in the cohort without clinically significant increases in neurotoxicity [122].

### Safety analyses

Retrospective safety analyses in patients with advanced solid tumors receiving RT and PD-1/PD-L1 and/or CTLA-4 blockade have generally not demonstrated increased risk of toxicity with the combination beyond those expected with each modality independently [123, 124]. There were no significant differences in toxicity rates between choice of PD-1/PD-L1 and CTLA-4 inhibitor or concurrent and sequential treatment with RT [124]. However, another series of 29 metastatic NSCLC patients given thoracic RT and PD-1/PD-L1 and/or CTLA-4 inhibitors identified 1 case of possibly treatment-related grade 5 pneumonitis in a patient who received 20 Gy over 5 fractions of thoracic RT initiated 1 month after the last dose of anti-PD-1 therapy [125]. Interestingly, case reports have documented the existence of PD-1 inhibitor-induced radiation recall pneumonitis even after 2 years of RT [126].

A multicenter safety analysis demonstrated no significant differences in immune-related AEs regardless of site of irradiation, between those receiving RT during/after checkpoint inhibitors and before checkpoint inhibitors (*p* = 0.053), and between those receiving RT within 14 days or outside 14 days of checkpoint blockade (*p* = 0.06) [127]. One retrospective series demonstrated that brain RT and PD-1/PD-L1 blockade was relatively well-tolerated in patients with NSCLC brain metastases as toxicity rates were consistent with those seen with checkpoint inhibitors alone [128]. Interestingly, the distant brain control (out-of-field) rate for RT during/before PD-1/PD-L1 blockade was 57% compared to 0% (RT after, *p* = 0.05). Another retrospective series of 137 patients with melanoma brain metastases identified 37 patients (27%) who developed radionecrosis following SRS or WBRT and anti-CTLA-4 or anti-PD-1 therapy with a median time of onset of 6 months (range 1.3–31.4 months), which is comparable to rates seen in other series though prospective studies are limited [129–132]. Notably, 1-year OS did not significantly differ between those that developed radionecrosis vs. those without (Table 2). However, risk of radionecrosis was significantly associated with concurrent use of chemotherapy within 6 months of SRS (HR 2.20, 95% CI 1.22–3.97, *p* = 0.009) and increased number of lesions treated (HR 1.09, 95% CI 1.03–1.15, *p* = 0.002). The lack of significant difference in OS between presence and absence of radionecrosis conflicts with the results of other studies though the number of patients treated with brain RT and PD-1 blockade were likely much smaller [130, 133].

### Prospective studies

A combined preclinical and phase I study was among the first to provide preliminary results for the efficacy of combined RT and checkpoint blockade in the prospective setting [134]. In the phase I dose-finding cohort of 5 patients given local RT for mixed response or asymptomatic progression to atezolizumab, dual RT and anti-PD-L1 therapy was well-tolerated without any dose-limiting toxicities (DLTs) or severe immune-mediated AEs and all 5 patients experienced at least SD (Table 3).

**Table 3:** Prospective clinical studies with available results on the antitumor activity of combined radiation therapy and PD-1/PD-L1 blockade

Study	n	Design	Outcomes	Toxicities	Ref.
Phase I	4 solid tumors, 1 hematologic malignancy	Atezolizumab 0.01–20 mg/kg every 3 weeks (dose-finding cohort) + local fractionated RT (dose NR) for mixed responses or asymptomatic PD	Stabilization of systemic progression in all 5 patients (PR at systemic site in 1 patient)	Transient grade 1–2 inflammatory AEs (fevers, flu-like symptoms) observed but no DLTs or serious immune-related AEs	[134]
Phase I	9 advanced melanoma	Nivolumab 0.3–10 mg/kg every 3 weeks X 21 weeks (induction) then every 12 weeks X 84 weeks (maintenance) ± ipilimumab 3 or 10 mg/kg every 3 weeks X 9 weeks (induction) then every 12 weeks X 84 weeks (maintenance) or combined nivolumab 1 mg/kg and ipilimumab 3 mg/kg every 3 weeks X 12 weeks then nivolumab 3 mg/kg every 2 weeks up to 96 weeks + RT (median 30 Gy X 5 fractions, range 21–37.5 Gy X 1–15 fractions) during induction or maintenance	ORR 44% (4 PRs) as best response by WHO criteria; median OS 27 mo; 1- and 2-year OS rates of 89 and 78%, respectively	5 patients with non-laboratory grade ≥ 3 AEs, 2 RT-related grade ≥ 3 AEs (intracranial hemorrhage, diarrhea)	[135]
Phase I/II	10 unresectable or metastatic solid tumors (≥5% PD-L1 expression)	Durvalumab 10 mg/kg every 2 weeks + local RT (median 20 Gy, range 6–33 X median 5 fractions, range 1–10) given a median of 8.5 days (range 1–35) of last dose of durvalumab	In-field ORR 60% (2/10 CRs, 4/10 PRs); median OS 11.5 mo (95% CI 8.8–13.7); median PSF 6.2 months (95% CI 4.5–12.4); out-of-field 10/14 SD, no responses or abscopal effects were seen	5 cases of (50%) RT-related grade 2 AEs (3 mucositis, 1 diarrhea, 1 vomiting)	[136]
Phase I	24 metastatic pancreatic adenocarcinoma	SBRT (8 Gy X 1 fraction or 25 Gy X 25 fractions) + durvalumab (dose NR) every 2 weeks or tremelimumab (dose NR) every 4 weeks X 6 doses then every 12 weeks for 3 doses or triple therapy	SD as best ORR in 5 patients (21%)	No DLTs observed; most common AE was grade 1–2 fatigue at dose level 2	[137]
Phase II	10 locally advanced NSCLC	Weekly carboplatin (AUC 2) and weekly paclitaxel 50 mg/m^2^ + RT 5 days/week for 6–7 weeks (60–66 Gy over 30–33 fractions) followed by atezolizumab 1200 mg every 3 weeks + consolidation carboplatin (AUC 6) and paclitaxel 200 mg/m^2^ on days 1 and 22 for 2 cycles then atezolizumab alone up to 1 year	Out of 7 patients receiving atezolizumab, 2 patients developed PD after 6 and 8 doses of atezolizumab	3 patients with potential immune-related AEs (1 grade 3 arthralgia, 1 grade 2 pneumonitis resolved with steroids, 1 grade 3 dyspnea)	[138]
Phase III	709 stage III, locally advanced, unresectable NSCLC	2 or more cycles of platinum-based chemotherapy (defined by local practice) + concurrent definitive RT (54–66 Gy with mean dose to the lung < 20 Gy or volume of lung parenchyma receiving ≥20 Gy < 35%) followed by (within 1–42 days) durvalumab 10 mg/kg every 2 weeks up to 1 year or placebo if no PD during chemoradiation	Median PFS 16.8 months (95% CI 13.0–18.1) vs. 5.6 months (95% CI 4.6–7.8) with placebo (HR 0.52, 95% CI 0.42–0.65, *p* < 0.001); median TTD or distant metastasis 23.2 months (95% CI 23.2-NE) vs. 14.6 months (95% CI 10.6–18.6) with placebo (HR 0.52, 95% CI 0.39–0.69, *p* < 0.001); ORR 28.4% vs. 16.0% with placebo (*p* < 0.001)	Grade 3–4 AEs 29.9% vs. 26.1% (placebo); most common grade 3–4 AEs pneumonia (4.4% vs. 3.8%), pneumonitis (3.4% vs. 2.6%), and anemia (2.9% vs. 3.4%) in durvalumab vs. placebo arms	[139]

*RT* radiation therapy, *NR* not reported, *PD* progressive disease, *PR* partial response, *DLT* dose-limiting toxicity, *AEs* adverse events, *Gy* Gray, *ORR* overall response rate, *PR* partial response, *WHO* World Health Organization, *CI* confidence interval, *SD* stable disease, *SBRT* stereotactic body radiation therapy, *NSCLC* non-small cell lung cancer, *AUC* area under curve, *CR* complete response, *PFS* progression-free survival, *HR* hazard ratio, *TTD* time to death, *NE* not estimable or reached

In another phase I trial, 9 patients with advanced melanoma received RT during induction, between induction and maintenance, or during maintenance therapy with ipilimumab and/or nivolumab [135]. Combined RT and checkpoint inhibition resulted in SD or response by first assessment at all irradiated sites and the best ORR was 44% (4 patients with partial responses (PRs)) by World Health Organization (WHO) criteria (Table 3). A phase I/II study investigated the safety and efficacy of concurrent local palliative RT and durvalumab (PD-L1 inhibitor) in 10 patients with unresectable or metastatic advanced solid tumors [136]. When RT (to 15 localized lesions) was given a median of 8.5 days (range 1–35) from the last dose of durvalumab, the combination was generally tolerated with no grade ≥ 3 RT-related AEs (Table 3). The 1-year OS and progression-free survival (PFS) rates were 44% (95% CI 12–77) and 30% (95% CI 2–58), respectively.

Preliminary results from a phase I dose-finding study of stereotactic body RT (SBRT; 8 Gy X 1 or 5 Gy X 5) and durvalumab or the CTLA-4 inhibitor tremelimumab (or combination of all 3) was administered as second-line therapy to 24 metastatic pancreatic adenocarcinoma patients. No DLTs have been observed so far [137]. The best response was SD in 5 patients (21%) with rapid progression within 4 weeks in an additional 5 patients. A phase II trial involving locally advanced NSCLC patients recently reported preliminary results from part I of the study [138]. Out of 10 enrolled patients, 7 have received atezolizumab added to consolidation carboplatin and paclitaxel following weekly carboplatin/paclitaxel and RT and 2 patients have demonstrated PD after 6 and 8 doses of the PD-L1 inhibitor. Given the safety and tolerability of patients in part I, criteria were met for advancement to part II of the study where atezolizumab will be added to the chemoradiation portion followed by consolidation atezolizumab, carboplatin, and paclitaxel.

Recently, the PD-L1 inhibitor durvalumab was granted FDA approval based on superior PFS but similar safety compared to placebo following platinum-based chemoradiation in locally advanced, unresectable NSCLC in the phase III PACIFIC trial [139]. Patients who did not demonstrate PD after ≥2 cycles of platinum-based chemotherapy concurrent with definitive RT were administered durvalumab or placebo within 1–42 days for up to 1 year (Table 3). Improved outcomes were observed in the experimental arm irrespective of PD-L1 status or histology.

## Discussion

Elucidated mechanisms underlying the immune stimulatory properties of RT are growing in complexity (Fig. 1). The CD8+ T-cell remains a crucial component in the ability of RT to elicit an antitumor immune response within and beyond the radiation field [140]. In addition, evidence is mounting to support that RT specifically upregulates MHC tumor-associated antigen complexes, enhances tumor antigen cross-presentation in draining lymph nodes, and increases T-cell infiltration into tumors [79, 141]. Local RT appears necessary in eliciting abscopal effects, but RT alone remains insufficient in complete eradication of local and distant tumors likely, in part, due to activation of negative T-cell regulatory pathways including the PD-1/PD-L1 axis and immune checkpoints such as CTLA-4 [76, 86, 87]. However, RT has been shown to upregulate expression of PD-1 and PD-L1 on immune and tumor cells rendering it an attractive modality to combine with PD-1/PD-L1 blockade [71, 76, 78, 79, 86, 97]. Activation of cGAS-STING signaling has also been recognized to mediate systemic tumor rejection by combined RT and checkpoint blockade given that knockdown of cGAS and STING in cancer cells abrogated priming of CD8+ T-cells in tumor-draining sites and infiltration of abscopal tumors by CD8+ T-cells [89].

In efforts to characterize the synergistic antitumor activity of combined RT and PD-1/PD-L1 blockade, numerous studies have identified significant elevations in CD8+ IFNγ+ TNFα+ T-cells but decreases in CD4+ FOXP3+ Tregs leading to an increased CD8+/Treg ratio, increases in tumor-antigen specific CD8+ TILs with a CD44+ effector memory phenotype, decreases in immunosuppressive MDSCs, reinvigoration of CD8+ TILs with an exhausted phenotype, and increases in TCR repertoire clonality and diversity of the TCR repertoire in irradiated and out-of-field sites as a consequence of combination radioimmunotherapy [61, 72, 76, 79, 88]. Furthermore, addition of anti-PD-L1 therapy to tumors that are nonresponsive to RT has shown the ability to reverse RT-induced tumor equilibrium in favor of tumor regression [92]. Resistance to RT also appears to be regulated by host STING activation via CCR2; additional targeting of the CCR2 pathway may therefore aid in reversing RT resistance in the context of checkpoint blockade [93]. Conversely, integration of RT to anti-PD-1-resistant tumors restores response to PD-1 blockade highlighted by RT-induced IFN-γ production and MHC class I expression [91].

Immune modulation from immune checkpoint inhibitors and RT through nonredundant pathways that altogether contribute to synergistic antitumor activity now represents an emerging theme from ongoing investigations in combination RT and immunotherapy [61, 77, 85, 88, 90, 142]. For example, anti-CTLA-4 therapy has been shown to predominantly inhibit Tregs, increase the CD8+ T-cell/Treg ratio, and promote T-cell expansion. Radiation enhances the diversity of the TCR repertoire, shapes the TCR repertoire of expanded peripheral T-cell clones in an antigen-driven selection manner, and promotes tumor infiltration by antigen-specific CD8+ T-cells. Addition of PD-1/PD-L1 blockade reverses T-cell exhaustion to offset decreases in the CD8+ T-cell/Treg ratio and further enhances oligoclonal T-cell proliferation.

Several points of consideration remain that could potentially impact the rational combination of RT and PD-1/PD-L1 inhibitors and their efficacy. Firstly, immunogenic cell death has been shown to be induced by RT in a dose-dependent manner in vitro [68]. In other preclinical studies, increasing radiation doses (single fractions above 7.5 Gy but not 5 Gy) were immunostimulatory, associated with elevated IFN-γ production, and prevented increases in Tregs [143]. At higher doses (single fractions ≥15 Gy), dose-dependent increases in Tregs were observed and associated with no improvement in antitumor immune responses. Fractionation of the 15 Gy generally resulted in superior immune responses compared to single-fraction 15 Gy. In a seminal study of 2 preclinical mouse carcinoma models, evaluation of RT (20 Gy X 1, 8 Gy X 3, or 6 Gy X 5 fractions over consecutive days) in combination with an anti-CTLA-4 antibody determined that fractionated RT but not single-dose RT achieved significantly enhanced tumor responses both within and outside the radiation field (abscopal effects) when combined with CTLA-4 blockade [55]. It has been further corroborated that fractionated RT (8 Gy X 3) with checkpoint blockade was able to elicit abscopal effects whereas checkpoint blockade with RT doses ≥20 Gy single dose were characterized by complete loss of abscopal responses through induction of Trex1 and downregulation of type I IFN signaling [89].

The timing of RT in relation to administration of checkpoint inhibitors represents another issue of discussion. Preclinical data support that RT-associated increases in the CD8+ T-cell/Treg ratio, CD8+ T-cell PD-1 expression, and tumor cell PD-L1 expression often occur early with peak levels occurring within 24–96 h post-RT [81, 86]. In an elegant study exploring combined anti-PD-L1 therapy and fractionated RT (10 Gy in 5 daily fractions), the addition of PD-L1 blockade on day 1 of RT (concurrent regimen starting at the beginning of RT), day 5 of RT (concurrent regimen starting at the end of RT), or 7 days after RT (sequential therapy) showed that there was no significant difference in OS between either concurrent therapy schedules [86]. However, sequential therapy was ineffective in enhancing OS compared to RT alone (median OS 30 days vs. 35 days, *p* > 0.05). Interestingly, the rise in PD-1 expression on CD8+ T-cells was evident up to 7 days after the last dose of RT, after which PD-1 levels significantly decreased compared to time-matched controls. In the clinical setting, retrospective series have documented a wider range of schedules in combining radioimmunotherapy ranging from RT at any point prior to immune checkpoint therapy, within 1 month of administration of checkpoint inhibitors, or up to 1 year of checkpoint blockade [117, 121, 124, 129]. Moreover, results have been largely mixed on the impact of scheduling of RT and checkpoint blockade on survival as several retrospective studies have identified that there is no significant difference in OS between concurrent and nonconcurrrent radioimmunotherapy while another study demonstrated a significant improvement in PFS and OS in patients having ever received RT prior to PD-1 blockade compared to those with no prior RT [116, 117, 121]. It is worthwhile to mention that these retrospective studies were likely limited by variability in RT modality, tumor histology, patient characteristics, and cohort size. Notably, abscopal effects have been observed in 56% of patients with the addition of late RT to PD-1 blockade as well (> 3 months of insufficient response to anti-PD-1 monotherapy) [119].

Another point of consideration in clinical trial design is the issue of toxicity with combined RT and PD-1/PD-L1 blockade. Several preclinical studies demonstrated more findings of abnormal alveoli, inflammatory changes, exudates in the alveolar septa, and cardiac toxicity in mice receiving thoracic RT and anti-PD-1 therapy, when compared to controls, though effects on survival have been mixed [100–102]. Retrospective analyses have generally shown no increased risk of toxicity with the combination of RT and checkpoint blockade beyond those expected with either modality alone [121, 124, 127]. For brain RT, a study of 137 patients treated with SRS or WBRT in combination with PD-1 or CTLA-4 blockade identified radionecrosis in 27% though 1-year OS did not significantly differ between those that developed radionecrosis and those that did not [129]. Reassuringly, retrospective series of > 200 patients receiving combined RT and immunotherapy have demonstrated that there are no significant differences in toxicities regardless of site of irradiation, choice of checkpoint inhibitor, or treatment schedule (concurrent vs. sequential) [124, 127].

Taking together the preclinical evidence on the kinetics of PD-1 and PD-L1 expression in relation to RT and the clinical data on the safety and tolerability of radioimmunotherapy, there is growing evidence to support that PD-1/PD-L1 blockade is optimal when synchronized with the administration of fractionated RT to prevent the development of immunological anergy [144]. Indeed, the concept of administering PD-1/PD-L1 inhibitors concurrently or immediately following fractionated RT has already been employed in clinical trials with evidence that the combination is generally well-tolerated (Table 3). However, despite our increased understanding, preclinical and clinical data have yet to offer a consensus on optimal dosing and modality sequencing to date [68]. The majority of retrospective and prospective studies on combination RT and checkpoint blockade have predominantly used fractionated dosing schemes (Tables 2 and 3). However, depending on the tumor type, target site, and modality employed, total RT doses from retrospective series have ranged widely from 8 to 74 Gy (Table 2). Of the limited number of larger prospective trials, PD-1 and PD-L1 blockade have often been incorporated into standard dosing regimens of SBRT and chemoradiation routinely used in the treatment of locally advanced pancreatic cancer and NSCLC, for example (Table 3).

It is worthwhile to mention that the Phase III PACIFIC trial demonstrated the superiority of chemoradiation followed by durvalumab when the latter was included within 1–42 days of chemoradiation over chemoradiation followed by placebo in locally advanced NSCLC [139]. On review of the study protocol and Supplementary Appendix, the investigators emphasized the initiation of durvalumab as close as possible to chemoradiation when antigen release and PD-L1 expression is likely to be at its greatest. An analysis of benefit in those receiving durvalumab closer to chemoradiation compared to those treated later relative to chemoradiation was not provided; an analysis of this nature may provide further insight on the proposed synergism offered by this combination. For reasons which are unclear, the median PFS of the placebo arm (5.6 months) appears worse than historical standards [145]. It is also unclear whether the benefit derived from the combination arm is due to the efficacy of immunotherapy in settings of smaller disease volume as seen previously [146]. All of these are potential factors that may contribute to the difference seen in efficacy between experimental and control arms.

Despite the promising results and feasibility of the PACIFIC trial, clinical studies on an upper threshold RT dose with checkpoint inhibition by which no further improvement in antitumor immunity is offered (as foreshadowed by preclinical evidence discussed previously) are virtually nonexistent, yet duly warranted. Dedicated dose-escalation studies on combination PD-1/PD-L1 inhibitors and RT are also needed in other tumor types to determine safety and tolerability. Early phase studies of this nature are emerging and have demonstrated the feasibility of this combination while recognizing the importance of timing of checkpoint blockade with respect to RT administration [147]. Extrapolation of RT dose effects from animal to human studies is not straightforward and great caution is needed in applying dosing schemes and regimens involving combination RT and PD-1/PD-L1 blockade in human patients [148]. Further understanding of the mechanistic and dynamic immunostimulatory properties of RT and PD-1/PD-L1 blockade are undoubtedly warranted with validation in (ideally) prospective cohorts prior to maximizing tumor responses with the combination. The ability to optimize immune responses in the future with radioimmunotherapy may potentially depend on the immunotherapeutic strategy used, tumor histology, balance between proimmunogenic and immunosuppressive effects of either modality, and other host factors [50, 148].

Lastly, phase I trials of RT and anti-PD-1 therapy have already provided glimpses into potential mechanisms of failure even with the combination as 1 patient with metastatic RCC who rapidly progressed on combined RT and pembrolizumab had biomarker analyses showing an absence of TILs and presence of other nonredundant immune checkpoints in the tumor microenvironment and periphery that may have contributed to treatment failure [149]. Accordingly, future studies may seek to target multiple checkpoints in combination with RT. The incorporation of additional immunotherapeutic strategies or other systemic therapies to enhance immune responses with RT represents another potential avenue of therapy. Several studies have investigated combined RT, PD-1/PD-L1, and CTLA-4 blockade while others have evaluated RT and immune checkpoint therapy with various combinations of chemotherapy, vaccine therapies, or targeted therapies across a spectrum of cancers [150–157].

## Abbreviations

AEs: Adverse events
CCR2: Chemokine receptor type 2
cGAS: Cyclic GMP-AMP (cGAMP) synthase
CI: Confidence interval
CR: Complete response
CTLA-4: Cytotoxic T-lymphocyte antigen 4
DLTs: Dose-limiting toxicities
FDA: Food and drug administration
GEMM: Genetically engineered mouse model
Gy: Gray
HCC: Hepatocellular carcinoma
HR: Hazard ratio
HSCT: Hematopoietic stem cell transplantation
IFNγ: Interferon-γ
IQR: Interquartile range
LAG3: Lymphocyte activation gene 3 protein
MDSCs: Myeloid-derived suppressor cells
MHC: Major histocompatibility complex
NSCLC: Non-small cell lung cancer
OS: Overall survival
PD-1: Programmed cell death 1
PD-L1: Programmed death-ligand 1
PFS: Progression-free survival
PR: Partial response
RCC: Renal cell carcinoma
RFA: Radiofrequency ablation
RT: Radiation therapy
SABR: Stereotactic ablative radiotherapy
SD: Stable disease
SRS: Stereotactic radiosurgery
STING: Stimulator of interferon genes
TAMs: Tumor-associated macrophages
TBI: Total body irradiation
TCR: T-cell receptor
TILs: Tumor-infiltrating lymphocytes
TIM-3: T-cell immunoglobulin mucin-3
TNFα: Tumor necrosis factor-α
Tregs: Regulatory T-cells
WBRT: Whole brain radiotherapy
WHO: World Health Organization
WT: Wild-type
